# A deep learning approach for identifying cancer survivors living with post-traumatic stress disorder on Twitter

**DOI:** 10.1186/s12911-020-01272-1

**Published:** 2020-12-14

**Authors:** Nur Hafieza Ismail, Ninghao Liu, Mengnan Du, Zhe He, Xia Hu

**Affiliations:** 1 Department of Computer Science & Engineering, Texas A&M University, College Station, TX USA; 2 School of Information, Florida State University, Tallahassee, FL USA

**Keywords:** PTSD, Cancer survivor, Social media, Deep learning

## Abstract

**Background:**

Emotions after surviving cancer can be complicated. The survivors may have gained new strength to continue life, but some of them may begin to deal with complicated feelings and emotional stress due to trauma and fear of cancer recurrence. The widespread use of Twitter for socializing has been the alternative medium for data collection compared to traditional studies of mental health, which primarily depend on information taken from medical staff with their consent. These social media data, to a certain extent, reflect the users’ psychological state. However, Twitter also contains a mix of noisy and genuine tweets. The process of manually identifying genuine tweets is expensive and time-consuming.

**Methods:**

We stream the data using cancer as a keyword to filter the tweets with cancer-free and use post-traumatic stress disorder (PTSD) related keywords to reduce the time spent on the annotation task. Convolutional Neural Network (CNN) learns the representations of the input to identify cancer survivors with PTSD.

**Results:**

The results present that the proposed CNN can effectively identify cancer survivors with PTSD. The experiments on real-world datasets show that our model outperforms the baselines and correctly classifies the new tweets.

**Conclusions:**

PTSD is one of the severe anxiety disorders that could affect individuals who are exposed to traumatic events, including cancer. Cancer survivors are at risk of short-term or long-term effects on physical and psycho-social well-being. Therefore, the evaluation and treatment of PTSD are essential parts of cancer survivorship care. It will act as an alarming system by detecting the PTSD presence based on users’ postings on Twitter.

## Background

PTSD is a psychological disorder that occurs in some people after witnessing or experiencing traumatic events [1]. People who have suffered from war, a severe accident, a natural disaster, a sexual assault, and medical trauma are potentially at risk of developing PTSD. Almost half of the cancer fighters are diagnosed with a psychiatric disorder, with the majority of them having chronic depression [2]. Cancer diagnosis, treatments (chemotherapy and radiation), post-treatment care, and recovery could affect the patients’ psychological condition and cause anxiety or trauma. Unstable mental health among cancer survivors is hazardous because they are at high risk of self-destruction and may also harm others once they lose self-control of their behaviors [3].

The diagnostic procedure for mental illnesses is different from physical illnesses. Traditional mental illness diagnosis begins with patients’ self-reporting about unusual feelings and caregivers’ perception of the patients’ behavior to the doctor. To diagnose a patient, a doctor will conduct a physical examination, order lab tests, and perform a psychological evaluation that requires a period of observation of the symptoms. The psychological assessment will be conducted by a psychiatrist who has an extensive breadth of knowledge and experience not only in mental health but also in general medicine. This process of making a diagnosis is not easy, and it takes a lot of time and effort to find effective treatments. Thus, in this work, we want to capture the presence of PTSD symptoms in cancer survivors from online social media postings so they can have an early meeting with a doctor and receive immediate treatment to calm the stress.

Information about the user’s online activities has been used to identify several health problems in the previous work [4]. The growth of social media sites in recent years has made it a promising information source for investigating issues on mental health. For example, Twitter has a large user base with hundreds of millions of active users [5]. It has simple features that allow users to share their daily thoughts and feelings [6]. The online activities, especially postings on the timeline, may present an insight into emotional crash towards significant incidents that happened in life. Related studies have shown the potential of Twitter for detecting the early symptoms of mental illnesses [7].

Previous work in mental illness on social media aimed to examine the attitude of self- declared mentally ill patients based on their interactions with others and social aspects from their written comments and postings [8, 9]. The study conducted by De Choudhury et al. [7] used crowd-sourcing to access Twitter users who have been diagnosed with major depressive disorder by a psychiatrist. The Linguistic Inquiry Word Count (LIWC) was used to characterize linguistic styles in tweets. Most of the previous studies only focus on identifying mental illnesses in social media users generally.

In addition, some experimental procedures such as crowd-sourcing, Twitter Firehouse, manual labeling, and LIWC are expensive and time-consuming. Data collection, data pre-processing, and analysis are challenging due to the following reasons. First, there are no available techniques that can verify if a tweet contains elements about both cancer-free and PTSD. Second, the extracted information related to mental health is not fully utilized in developing psychological screening tools for cancer survivors.

To tackle these challenges, we propose a technique to classify cancer survivor and PTSD related tweets. To identify PTSD in cancer survivors, we first crawl the tweets using ‘cancer’ as a keyword. After that, we use a set of cancer survivor and PTSD keywords to filter out irrelevant tweets, which can reduce the time required for manual labeling. To create a ground truth dataset for this work, we make an effort to check the extracted tweets again manually. The primary purpose of the manual checkup is to make sure that the extracted tweets are correctly labeled as to whether the tweet contained a genuine statement of a cancer survivor with PTSD diagnosis. In this work, we used the Deep Neural Network (DNN) approach that learns to extract meaningful representations of texts and identify key features from the input dataset. The conference version of this work was previously published in [10]. More technical details on model derivation, applications, and experimental evaluations are provided in this extended paper. Specifically, we will answer the following research questions (RQs). RQ1: How to capture tweets that contain characteristics of cancer survivors living with PTSD (ground truth data collection)? RQ2: How to incorporate tweets into a deep-learning-based framework to construct a prediction model of cancer survivors living with PTSD? RQ3: How reliable is the extracted model for tweet classification of cancer survivors living with PTSD? Following these RQs, we present a framework that can automatically identify PTSD from cancer survivors based on their tweets. The major contributions of this work are summarized as follows:

- We formally define the problem of data crawling and extracting techniques for retrieving the tweets that represent the cancer survivors with PTSD.
- We present a framework and training the proposed CNN to identify cancer survivors living with PTSD based on phrases on Twitter.
- We evaluate the model’s prediction performance by producing a label with associated probability for new tweets.

## Related work

Researchers from diverse backgrounds, such as psychology and medical informatics, have proposed early models for detecting mental health issues. They explored different types of datasets, feature extraction approaches, and modeling methods to develop a reliable model. The physiological features, such as facial expression, vocal acoustic, blood flow, and nervous system responses can indicate the presence of a person’s current emotions [11]. Various sensor measurements in medical examination results such as electrocardiography (ECG), electroencephalography (EEG), electromyography (EMG), functional magnetic resonance imaging (fMRI), and respiratory transducer have been used to identify emotional changes in PTSD diagnosis [12]. Besides, some experts also considered speech audio, interview video, and questionnaire, in both formal and informal ways [13, 14]. Nevertheless, collecting this information with these techniques is time-consuming and labor-intensive.

The alternative approach is to crawl the public online postings on social media, which are accessible, expeditious, and provides boundless access to a broader population. Almost 60% of adults use online resources for searching and sharing information about health [15]. Compared to asking doctors or friends, people feel more open to communicate and ask questions on social media. They can also have a conversation with people with a similar background and those who are currently facing the same health concern on the forums. Previous work has shown that text posts, votes, and comments on Reddit, a popular online discussion board, can reveal early symptoms of mental health conditions [15]. In a previous study on public health, Paul and Dredze [6] showed the linguistic style of the users from their tweets. These previous studies have motivated us to use Twitter data to grasp the implicit and explicit information behind the language used by PTSD patients with a cancer history.

Cured cancer patients are often concerned about cancer recurrence, which can be even more stressful and upsetting compared to first-time diagnosis [16]. Patients reported that it is harder to decide the treatment, the side effects are more serious, and the fears of pain increase [17]. This psychological impact that may lead to PTSD problems is one of the most significant concerns in clinical oncology [18]. Receiving immediate attention to PTSD can help to improve the quality of life. Nevertheless, the lack of quantifiable data for PTSD is one of the main obstacles for making reliable diagnoses and providing effective treatment [19]. These issues have been our second research motivation to collect data for cancer survivors living with PTSD.

There are several techniques applied to uncover essential features from mental health datasets. Commonly, medical experts who conduct similar research analyze the collected dataset using statistical methods, such as t-test, chi-square tests, correlations, linear regression, and logistic regression [20–22]. The dataset for mental health is gathered using a questionnaire to collect sociodemographic information, clinical variables, medical comorbidity, and self-reported depression to identify mental illness signs or symptoms. The analyses report the characteristics of each item in the percentage or scale value. From there, they can identify the most correlated factors for mental health diagnosis.

Numerous analytical methods and techniques, including supervised and unsupervised learning algorithms, have been applied for monitoring mental health symptoms. Regression analysis and the support vector machine [23–25], decision tree, and neural network [26] performed well with high diagnostic accuracy. For the unsupervised models, a linear discriminant analysis model can generate the topics found in engagement content on social media to investigate the engagement implication on mentally ill people [27]. DNN, a deep belief network model, was trained to extract PTSD features from a speech dataset using a transfer learning approach [28]. The DNN has shown promising results in Natural Language Processing (NLP). In this work, we developed a model using the DNN approach that can learn from different levels of representation of text input. This approach can learn from the input data and has been used widely to make predictions in various areas of automatic speech recognition, image recognition, and NLP. DNN automatically learns the representations from the input data and uses them for classification [29]. In comparison, traditional machine learning requires labor-intensive feature engineering that may result in a biased set of features.

## Methods

In this section, we will introduce the problem statements and the proposed framework, including feature extraction, knowledge transfer, and CNN architecture. Then, in the Experiments section, we will explain the data preparation process.

### Problem statement

#### Problem

We consider a relation exists in *n* tweets with *m* characteristics of cancer survivors living with PTSD. Each relation between a tweet *t*_*i*_ and characteristics *p*_*j*_ is represented as *e*_*ij*_ = (*t*_*i*_, *p*_*j*_). In particular, in our setting, a relation is composed of textual information related to the tweet *t*_*i*_ with characteristics of cancer survivors living with PTSD *p*_*j*_. Also, we assume that each tweet is associated with a label *L*(*t*_*i*_) = 1 if the tweet belongs to cancer survivor living with PTSD, otherwise *L*(*t*_*i*_) = 0. Throughout this paper, we will use italic characters *x* for scalars, bold characters h for vectors, and bold capital characters W for matrices.

#### Goal

We aim to actively explore the cancer survivors living with PTSD on Twitter. In particular, given a tweet containing characteristics of cancer survivors living with PTSD *E* = *{e*_*ij*_ = (*t*_*i*_, *p*_*j*_)*}*, our goal is to produce a prediction $\hat{L}\left({t}_i\right)\in \left[0,1\right]$ for each tweet and its probability score *s*_*i*_.

### The proposed framework for classifying tweets about cancer survivors living with PTSD

**Fig. 1:**
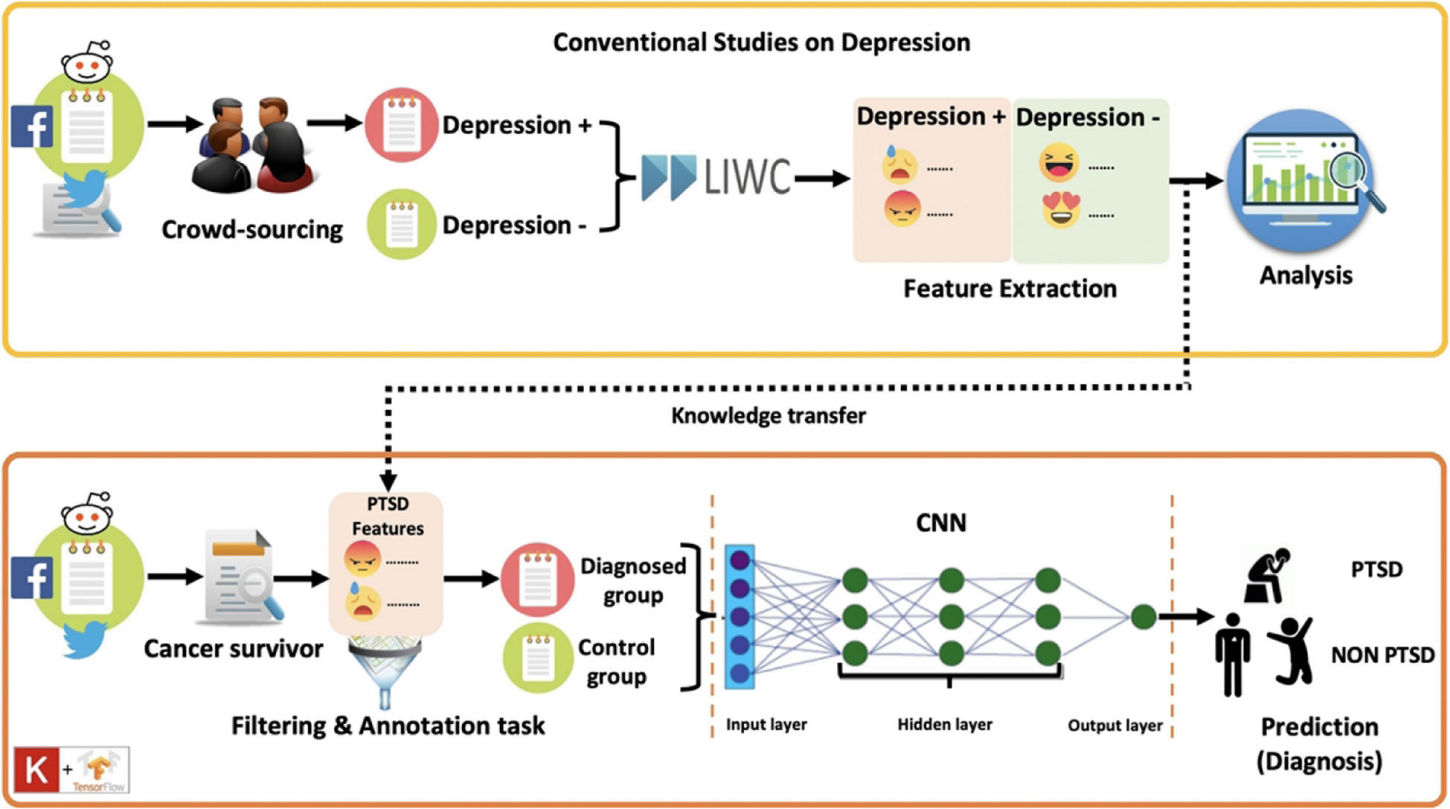
The overview of our proposed framework for classifying tweets about cancer survivors living with PTSD using CNN model

Figure 1 presents our proposed framework on classifying tweets about cancer survivors living with PTSD using CNN model. It involves two central parts. First, we extract a set of particular lexicons that are frequently mentioned by sufferers from previous studies on depression, which relates to PTSD. Second, the extracted lexicons are then used to capture tweets that contain PTSD symptoms in the cancer survivors dataset. The detailed process of our proposed framework will be explained in three subsections: (1) feature extraction, (2) knowledge transfer, and (3) CNN architecture.

#### Feature extraction

Feature extraction, also known as variable selection, aims to discover a small amount of valuable information that can best represent the whole large dataset. This process requires specific methods of extraction to be applied to the input data to create an accurate prediction model. The top section of Fig. 1 shows the overview of previous studies related to predicting depression on social media [7]. The crowd-sourcing approach has been employed to identify written postings, whether they are depression positive or depression negative.

Next, they used LIWC, a text analysis tool, to perceive the characteristics of linguistic style in both groups. It employed to count the psychological expression lexicon in the tweets and assess the proportions of words used in several linguistic categories. The given comprehensive list produced by the tool will automatically present the most frequently used lexicon by depressed people, and statistical methods are applied to visualize the analysis results. Table 1 shows the depression lexicon.

**Table 1:** The depression lexicon

Category	Unigrams
Symptoms	anxiety, withdrawal, severe, delusions, adhd, weight, insomnia, drowsiness, suicidal, appetite, dizziness, nausea, episodes, attacks, sleep, seizures, addictive, weaned, swings, dysfunction, blurred, irritability, headache, fatigue, imbalance, nervousness, psychosis, drowsy, PTSD
Disclosure	fun, play, helped, god, answer, wants, leave, beautiful, suffer, sorry, tolerance, agree, hate, helpful, haha, enjoy, social, talk, save, win, care, love, like, hold, cope, amazing, discuss
Treatment	medication, side-effects, doctor, doses, effective, prescribed, therapy, inhibitor, stimulant, antidepressant, patients, neurotransmitters, prescriptions, psychotherapy, diagnosis, clinical, pills, chemical, counteract, toxicity, hospitalization, sedative, drugs
Relationship and life	home, woman, she, him, girl, game, men, friends, sexual, boy, someone, movie, favorite, Jesus, house, music, religion, her, songs, party, bible, relationship, hell, young, style, church, lord, father, season, heaven, dating

#### Knowledge transfer

In this part, knowledge transfer can be interpreted as a task that uses depression lexicon in developing our PTSD positive dataset. This approach is similar to the transfer learning method in which the pre-trained models are used to reduce training time and to increase the performance of the model. Depression and PTSD often co-occur. Almost all PTSD patients also have a presence of depression in clinical and epidemiological samples. This co-occurrence reflects overlapping symptoms in both types of mental disorders [30]. The word ‘cancer’ is strongly correlated to negative emotions such as mortality, fear, and stigma [31]. The definition of PTSD in our context is a failure to recover from a traumatic event of cancer. Thus, any expression of negative sentiment related to cancer in a tweet posted by cancer survivor is considered as PTSD. Even though there is no existing PTSD lexicon available, we could use the depression lexicon as a proxy to remove irrelevant tweets. We first used the depression lexicon to labeled our ground truth. To ensure labelling accuracy, we manually reviewed these tweets to make sure they indeed represent cancer survivors with PTSD symptoms. Thus, we opted to utilize the depression lexicon taken from previous work to identify PTSD tweets to answer our RQ1.

The lower section of Fig. 1 presents our proposed framework to identify cancer survivors with PTSD. We crawled the raw dataset using ‘cancer’ as a keyword through Twitter’s Application Programming Interface (API) in a period of 3 months from August 2019–October 2019. We conducted the extraction process in two steps using two sets of keywords. First, we created the cancer survivor dataset using related hash-tags and terms such as ‘cancer survivor’, ‘cancer-free’, ‘I had cancer’, ‘post-cancer’, ‘survive from cancer’, and ‘free from cancer’. Second, we used the depression features from Table 1 to filter out tweets that are unrelated to PTSD signals. Next, in the annotation task, we checked the tweets manually to make sure the extracted tweets are correctly identified. The extraction process helped us save lots of time for the annotation task. The total data has decreased from 900,000 to only 5000 after we conducted the extraction process and the annotation task. Also, we added the word ‘PTSD’ in the Symptoms category to capture the word PTSD in tweets. Next, the extracted tweets were fed into CNN algorithms in the modeling phase.

### CNN architecture

**Fig. 2:**
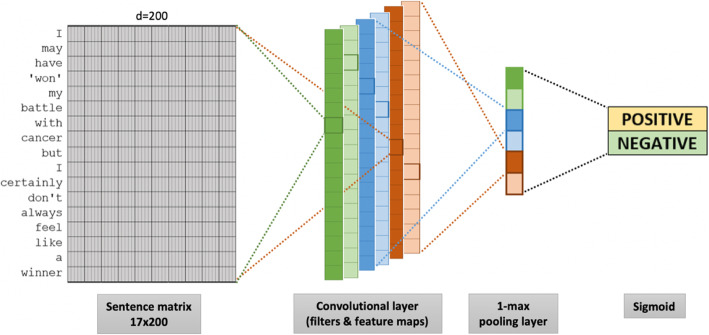
The CNN architecture to classify tweets posted by cancer survivors living with PTSD

The architecture of our proposed CNN model is inspired by [32] for sentiment analysis of the text. We adopted one convolutional layer during network configuration for cancer survivors with PTSD tweets classification, as displayed in Fig. 2. The specific CNN configuration for classifying cancer survivors living with PTSD based on tweets has answered our RQ2. We trained the CNN with the embedding layer. It requires specifying the vocabulary size, the size of the real-valued vector space, and the maximum length of words in input tweets. For convolutional feature maps, we used word embedding with 200-dimension for text representation. Thirty-two filters were applied by referring to the conservative setting for word processing, with a kernel size of 8, and with a Rectified Linear Unit (ReLU) activation function. Followed by a pooling layer, the filters will generate feature maps and reduce the output by half. The last layer uses a sigmoid activation function to output a boolean, i.e., positive and negative, in the tweets based on the concatenation of the previous vectors. Then, the extracted model is saved for later evaluation. The following subsections present the critical elements involved during network configuration.

#### Text representation

After the data cleaning process, we applied the embedding layer that is initialized with random weights. It learned an embedding for all of the words in the training dataset. The first step embeds the vocabulary file *V* to check the validity of the tokens in tweets.

Each input tweet is presented as a sequence of individual word tokens: [*t*_1_*,*. *.*.*, t*_*n*_] where *n* denotes the total number of tokens in the tweet. Tokens are represented by one-hot vectors **t** *∈ R*^1 *× d*^ to look up word embeddings **T** *∈ R*^*d × |V |*^. For every input tweet *s*, we built a string of words matrix **S** *∈ R*^*d × |s|*^, where every single column *i* represents a word embedding **t**_*i*_ of position *i* in a string. The CNN applies multiple configurations to the input string of words matrix **S** using convolution, non-linear activation, and pooling operations. It learns how to capture and to reconstruct features of individual tokens in a given tweet from word embeddings into higher-level concepts.

#### Convolutional feature maps

The purpose of the convolutional layer is to extract meaningful patterns from the input dataset using a number of filters. During convolutional operation, the input matrix **s** *∈ R*^1 *× |s|*^ and a filter **F** *∈ R*^*d × m*^ of the same dimensionality *d* with width *m* will produce a new vector of **c** *∈ R*^*|s|* + *m*1^, where each function is computed as follows:

1 $$ {\boldsymbol{c}}_i={\left(\boldsymbol{S}\ast \boldsymbol{F}\right)}_i=\sum \limits_{k,j}{\left({\boldsymbol{S}}_{\Big[i-m+1:i}\bigotimes \boldsymbol{F}\right)}_{kj,} $$

where *⊗* is the element-wise multiplication and **S**_[:*i − m* + 1:*i*]_ is a matrix slice with *m* size along with the columns. From Fig. 2, we can see that the filter overlays across the row vectors in the dimension table of *S*, producing a vector **c** *∈ R*^*|s| − m* + 1^ as the output. Each component *c*_*i*_ is the result of computing an element-wise product between a row slice of **S** and a filter matrix **F**, which is then summed up to obtain a single value. To grab more features and to form richer representation from the dataset, a series of filter **F** *∈ R*^*n × d × m*^ overlays the sentence matrix **S** and produces a feature map matrix **C** *∈ R*^*n × |s| − m* + 1^.

#### Activation functions

After the convolution step, we applied ReLU activation defined as *max*(0*,* **x**), which is the simplest non-linear activation function *α*() on the hidden layers. It has a lot of advantages. For example, it can generate a good result in a short time by reducing the training time for a large network.

#### Pooling

The output from the convolutional layer with ReLU activation function will be passed to the pooling layer. The goal of pooling is to control overfitting by combining the information and reducing the spatial size of the representation. In our model, we use *max* pooling to get the maximum value. It operates on columns of the feature map matrix C and returns the largest value: pool(**c**_*i*_): *R*^*|s|* + *mn −* 1^ *→ R*.

The convolutional layer utilizes the activation function, and the pooling layer acts as a non- linear feature extractor. Given that multiple feature maps are used in parallel to process the input, CNN can build rich feature representations of the data. The output of the convolutional and pooling layers are passed to a fully connected sigmoid layer. The main reason for using a sigmoid function is that it pushes the output to be between 0 and 1. Since the likelihood of any class exists only between the range of 0 and 1, sigmoid is appropriate for this setting.

### Experiment

We conducted the experiments to evaluate the proposed framework for classifying cancer survivors with PTSD diagnosis from tweets. First, we briefly describe the experiment setting and the dataset preparation process. Second, we introduce the baselines methods. Third, we report the experimental performances. Finally, we discuss our findings.

#### Experiment settings

In these experiments, the dataset with PTSD positive represents the diagnosed group, while PTSD negative represents the control group. For the diagnosed group, we retrieved tweets from users who publicly stated that they survived cancer and had PTSD symptoms. To construct the PTSD negative group, we mixed the tweets posted by cancer survivors with positive sentiment and tweets from the Kaggle dataset. We made use of tweets from the ‘Twitter User Gender Classification’ dataset from the Kaggle website.^1^

We used this dataset because we want to make sure that the PTSD negative dataset not only contains about cancer survivors with positive sentiment tweets but also other topics. Both groups have the same total number of 5 k tweets to create balanced datasets. The data preparation phase has three steps: (1) applying 5-fold cross-validation for Multiple Layer Perceptron (MLP), CNN, and CNN n-gram algorithms; applying Term Frequency–Inverse Document Frequency (TD-IDF) for Naive Bayes Classifier (NBC) and Support Vector Machine (SVM) algorithms (2) cleaning the dataset to remove punctuation, stop words, and numbers; (3) defining vocabulary of preferred words from a training dataset by stepping through words and keeping only tokens with minimum occurrences of five. This setting reduces the vocabulary size because we want to use only frequent tokens that appear in the dataset. We used Keras API running on Tensorflow to train DNN models. All the models were trained with ten epochs through the training data. The efficient Adam implementation of stochastic gradient descent was used. We keep track of performance in addition to loss during training. Table 2 shows the details of our CNN network setting.

**Table 2:** CNN network setting

Layer(type)	Output Shape	Param #
embedding 1& 2 (Embedding)	(None, 20, 200)	84,000
conv1d 1 (Conv1D)	(None, 13, 32)	51,232
max pooling1d 1& 2 (MaxPooling1D)	(None, 6, 32)	0
flatten 1 & 2 (Flatten)	(None, 192)	0
dense 1 (Dense)	(None, 10)	1930
dense 1 (Dense)	(None, 1)	11

### Baseline methods

We present the baseline methods used to evaluate our proposed algorithm. The input of our dataset was in a text format with positive and negative labels. Therefore, we chose four baselines that are capable of handling text dataset: NBC [33], SVM [34], MLP [35], and CNN n-gram [36]. NBC and SVM are considered as traditional machine learning algorithms. While MLP, CNN, and CNN n-gram are the DL algorithms.

#### NBC

NBC is based on the Bayes Theorem. For text classification, it will predict the membership probabilities for each class label, such as the probability that tweet belongs to a particular class label. The chosen class will have the highest probability value compared to other classes.

#### SVM

SVM is an algorithm that determines the best boundary between vectors that belong to a given group label and vectors that do not belong to the group. This technique can be applied to any vectors that encoded any data. Thus, for SVM text classification, we first must transform the texts into vectors.

#### MLP

The MLP is a feed-forward neural network that is frequently used for prediction models. The MLP used bag-of-words (BoW) to represent tweets. This technique can extract features from the text by measuring the occurrence of words within the documents. However, the BoW model suffers from sparse representation, which may have affected the space and time complexity. Moreover, it loses the semantics of the input sentences by ignoring the word order and grammar.

#### CNN n-gram

The kernel size in the convolutional layer defines the number of tokens that act as a group of the parameters. We set a model with two input channels for processing bi-grams and tri-grams of text in tweets due to the short length of words used in each tweet. This algorithm involves using multiple versions of the standard model with differently sized kernels for tweet classification. This setting allows tweets to be processed at a different number of contiguous words sequence, while the model learns how to integrate these interpretations best. The output from both channels was concatenated into a single vector and processed by a dense layer and an output layer.

## Results

### Experimental results

We ran the experiments using five different network settings. Our results indicate that CNN can effectively identify cancer survivors with PTSD. Experimental results in Table 3 show the 91.29% accuracy for CNN, which is higher than other baselines. We ran the experiments multiple times for MLP, CNN, and CNN n-gram algorithms due to the stochastic nature of DNN to get a reasonably accurate result.

**Table 3:** Experiment results of identifying cancer survivors with PTSD

Data Setting	Method	Accuracy	(%)
TD-IDF	NBC	86.5	
	SVM	49.0	
5-Fold Cross Validation	MLP	49.99	
	CNN n-gram	63.28	
	**CNN**	**91.29**	

**Fig. 3:**
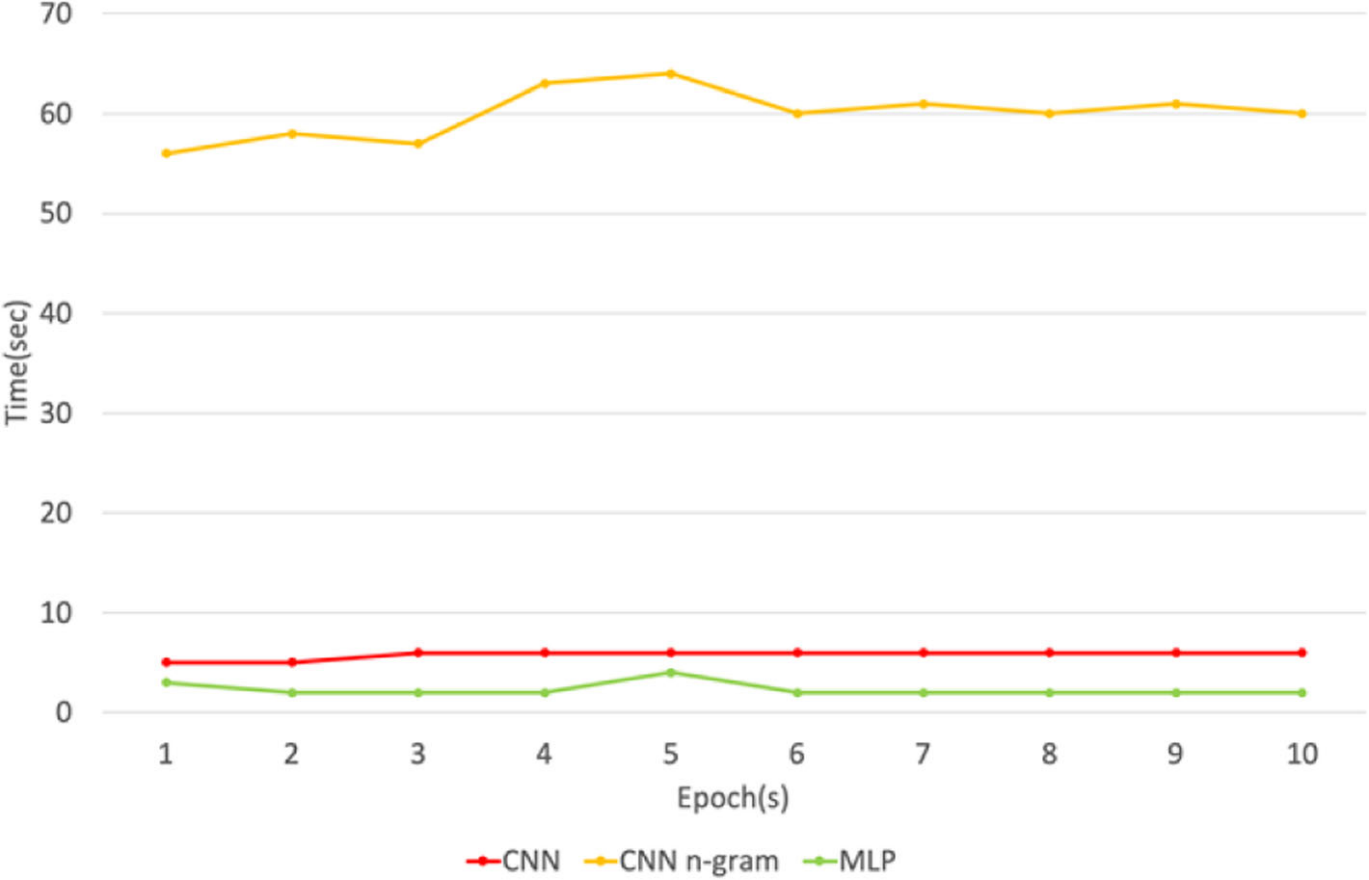
The learning time taken

**Fig. 4:**
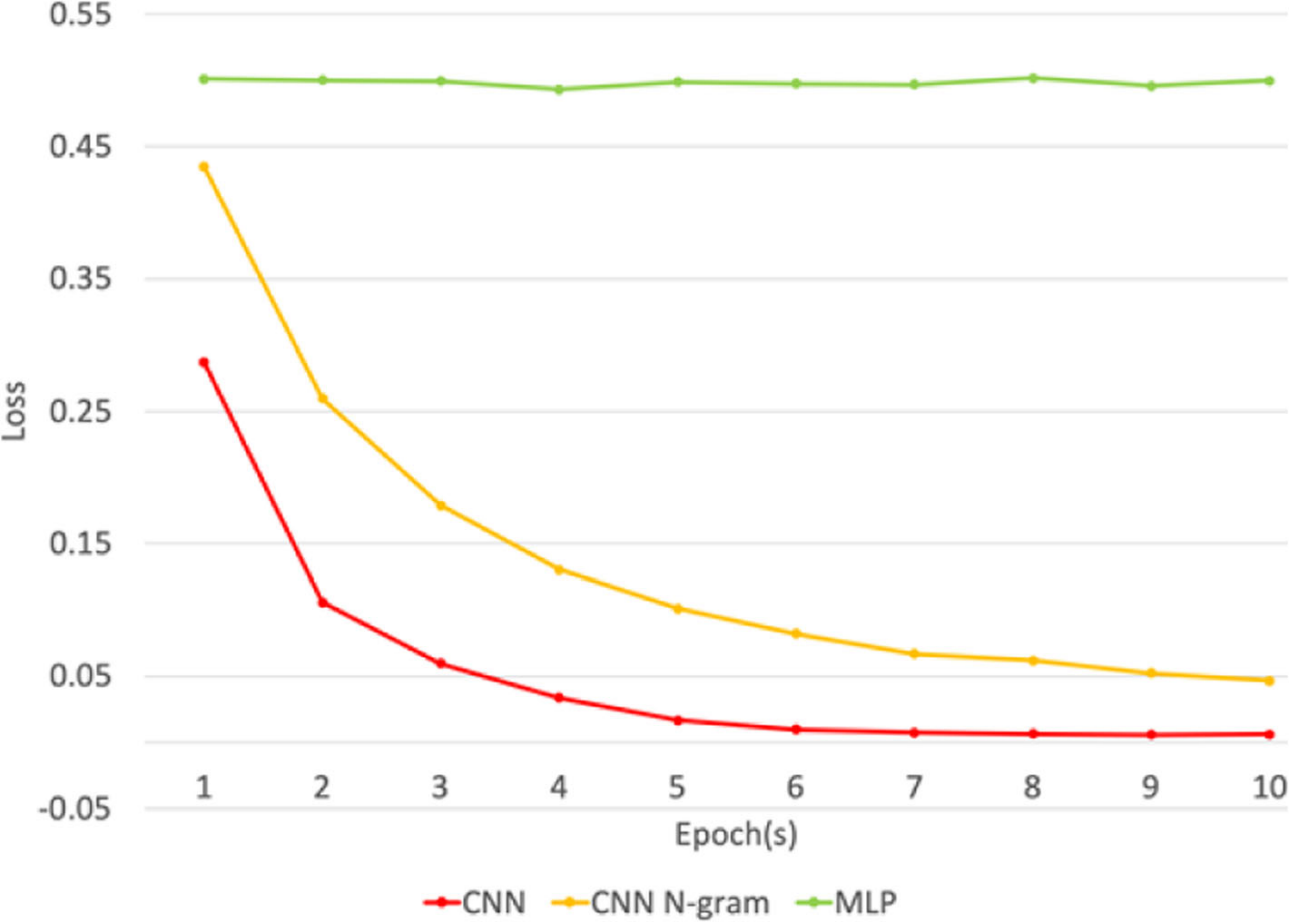
The loss values

Figure 3 presents the time taken during the DNN training process with MLP and CNN n-gram, which took slightly less time compared to CNN. Figure 4 shows the loss values in the training set of all models, where CNN and CNN n-gram display low losses. A model with the lowest loss value is better because loss value indicates errors made for examples during training. To test the CNN performance, we ran the experiment using only depression-lexicon as features. The experiment result is much worse, with 67.03% accuracy compared to our model. The results show that the model performed better with our set of vocabulary compared to a set of depression-lexicon taken from previous work. Even though we used depression-lexicon to help us to filter out unrelated tweets; however, our cancer survivor and PTSD tweets still contained unique characteristics and have different linguistic-style compared to depression users.

### Case study

We constructed a simple prediction system using the CNN model. It will identify cancer survivors as either PTSD positive or PTSD negative together with probability value on new tweets. The tweet samples and the output results are shown in Table 4. Surprisingly, the system was able to classify tweets correctly and has answered our RQ3. For example, the second tweet is a statement that consists of negative sentiment but not related to the cancer survivor, and the system classified it as a PTSD negative. The right labeling with high probability value is essential for the diagnosis.

**Table 4:** Predictions for new tweets

Tweet: “I have had more difficulty post cancer than during my active treatment. To me it is a neverending path (hate the word journey).” **POSITIVE (100.000%)**	
Tweet: “I hate myself, I don’t feel like living anymore.” **NEGATIVE (99.831%)**	
Tweet: “I got a gold MacBook that only use for music and homework. Still keep it in apple box.” **NEGATIVE (99.949%)**	

Meanwhile, Table 5 shows two examples of misclassified tweets. To test the model reliability, we replaced the word ‘cancer’ with ‘tumor’ and ‘cyst’, which are highly correlated to cancer. Unfortunately, the model failed to detect the presence of cancer-free and PTSD in both tweets. For example, the first tweet contains self-mention about having a bladder tumor and feel depressed, but our system classified it as a PTSD positive, which is wrong. This is because our model should detect the presence of PTSD symptoms in someone who is currently free from cancer. However, the prediction outcome that has a probability rate lower than 90% thus is less convincing and can be ignored for diagnosis.

**Table 5:** Predictions for new tweets (misclassified)

Tweet: “I have bladder tumor. I am totally heartbroken.” **POSITIVE (86.548%)**	
Tweet: “I am not a superwoman but I survived this pancreatic cyst. Time to enjoy with my family again!” **POSITIVE (86.548%)**	

Misclassified tweets may occur due to several reasons. First, it may be because the words ‘tumor’ and ‘cyst’ occasionally appeared in the dataset. Second, a small number of participants from this group were active in social media. To alleviate this problem, we need a larger dataset for training to leverage cancer-free with PTSD lexicon. Moreover, our model also should contain the information of diverse cancer types so the system will able to recognize them as a part of cancer rather than treating them as unknown words. To get special insights, we should make an effort to gather data from multiple sources. To the best of our knowledge, this is the first work that deployed the extracted model of cancer survivors living with PTSD into a prediction system that is capable of evaluating new tweets. The experimental results showed a high potential of a low-cost text classification technique that can be directly applied to other medical conditions that might affect patients’ mental health.

## Discussion and conclusions

PTSD is one of the severe anxiety disorders that could affect individuals who are exposed to traumatic events, including cancer. Cancer survivors are at risk of short-term or long-term effects on physical and psycho-social well-being. Therefore, the evaluation and treatment of PTSD are essential parts of cancer survivorship care. In this work, we demonstrated that Twitter could be used to identify PTSD among internet users who had cancer. We propose a prediction model that can produce promising results in cancer survivors with PTSD diagnosis. Experimental results demonstrated that CNN is capable of capturing important signals from texts. The social media users with cancer history who suffer from PTSD will benefit from the prediction system. It will act as an alarming system by detecting the PTSD presence based on users’ postings.

Essentially, we hope that our proposed data collection approach can facilitate current trauma screening questionnaire-based methods instead of replacing them. With the high rise of social media and a massive number of active users around the world, we hope to encourage more untreated cancer survivors that affected by PTSD to seek medical attention immediately. Moreover, the World Health Organization (WHO) stated that psychological disorder is the second largest of disability in the world population. However, only 10% of them obtained proper treatment.

Furthermore, we identify a cancer survivor who experienced PTSD only with one tweet. In this work, we did not use historical tweets because cancer is so daunting that some of the cancer survivors are even afraid to say ‘the C word’ [37]. Many aspects of cancer events can lead to PTSD, such as various diagnostic testing, stressful waiting periods, the moment of bad news, and the painful treatments. For cancer survivors, PTSD can be triggered by continuous monitoring, follow-up visits, sudden physical pain, death of a public figure due to cancer, and fear of cancer recurrence. The traumatic event of cancer might not be as clear as a life-threatening car crash, but it can completely change someone’s life. They may feel grief for possible lost future opportunities and may impact self-esteem because of disfigurements due to their disease. Because of that, we can spot tweets with negative sentiment related to cancer history when they express saddens, fear, stress, and enraged in their posting. Moreover, from our experience, when we went through their timeline, we noticed that they do not always express how they feel every day. This situation has made it hard for us to identify PTSD after cancer cases using historical tweets.

On the other hand, our model was trained to solely utilize the textual postings. The users’ contextual information, such as gender, ages, etc., is not considered in this work. To better improve our model in the future, additional main keywords that represent ‘cancer-free’ such as ‘cyst’ and ‘malignant tumor’ should be included during data crawling. From the case study, we can conclude that our proposed model cannot provide the right diagnosis when we replaced the word ‘cancer’ with ‘cyst’ and ‘tumor’ in the sentence. It is important because those words are highly correlated with ‘cancer’. Hence, we also want to identify developing conditions such as suicidal ideation and the side effect of PTSD treatment. Besides, we plan to explore another modality in uncovering PTSD indicators such as audio, image, or combination of both, for better diagnosis.

PTSD can also affect cancer survivors’ caregivers. Witnessing a loved one having cancer and watching the little one in pain are traumatic events that caregivers have to face. The Cancer. Net website reported that almost 20% of families of childhood cancer survivors had a parent who was suffering from PTSD. They also found that this anxiety disorder is common among parents of children receiving cancer treatment to develop PTSD symptoms. Thus, we believe that our work also can be utilized to identify PTSD in cancer survivors’ caregivers. However, we must formally define the problem and identify the implicit and explicit characteristics of caregivers because some of them may have a difficult time admitting they are depressed.

## Data Availability

The dataset used and analyzed during the current study are not publicly available due to negative emotions statements given by the cancer survivors on Twitter but are available from the corresponding author on reasonable request.

## Abbreviations

PTSD: Post-traumatic stress disorder
CNN: Convolutional Neural Network
LIWC: Linguistic Inquiry Word Count
DNN: Deep Neural Network
RQ: Research question
ECG: Electrocardiography
EEG: Electroencephalography
EMG: Electromyography
fMRI: Functional magnetic resonance imaging
NLP: Natural Language Processing
API: Application Programming Interface
ReLU: Rectified Linear Unit
MLP: Multiple Layer Perceptron
TD-IDF: Term Frequency–Inverse Document Frequency
NBC: Naive Bayes Classifier
SVM: Support vector machine
BoW: Bag-of-words
WHO: World Health Organization

## Footnotes

1 https://www.kaggle.com/crowdflower/twitter-user-gender-classification#gender-classifier-DFE-791531.csv
